# Production of White, Red and Black Quinoa (*Chenopodium quinoa* Willd Var. Real) Protein Isolates and Its Hydrolysates in Germinated and Non-Germinated Quinoa Samples and Antioxidant Activity Evaluation

**DOI:** 10.3390/plants8080257

**Published:** 2019-07-30

**Authors:** Lucrecia Piñuel, Patricia Boeri, Fanny Zubillaga, Daniel Alejandro Barrio, Joaquin Torreta, Andrea Cruz, Grace Vásquez, Adelita Pinto, Wilman Carrillo

**Affiliations:** 1 CIT-RIO NEGRO Sede Atlántica, Universidad Nacional de Rio Negro (UNRN-CONICET), Don Bosco y Leloir s/n, Rio Negro Viedma CP 8500, Argentina; 2 Research Department, Faculty of Health Sciences, Technical University of Babahoyo. Av. Universitaria Km 21/2 Av. Montalvo. Babahoyo CP 120301, Ecuador; 3 Faculty of Mechanical Engineering and Production Sciences, ESPOL Polytechnic University, Campus Gustavo Galindo Km 30.5 Vía Perimetral, P.O. Box 09-01-5863 Guayaquil, Ecuador

**Keywords:** quinoa, quinoa protein concentrates, antioxidant activity, hydrolysates, zebrafish embryos

## Abstract

Red, black and white seeds quinoa were germinated at 28 °C during 24 (G1), 48 and 72 h (G3). Red quinoa presented a higher percentage of germination with a value of 46% of germination at 72 h. Quinoa protein isolate (QPI) was obtained by alkaline extraction (pH 8.0) followed by an isoelectric precipitation (pH 4.5) from white, red and black quinoa seeds, germinated QPI-G1 or QPI-G3 and non-germinated QPI-NG, *Chenopodium quinoa* Willd var. Real. QPI-G1, QPI-G3 and QPI-NG were subject to a simulated gastric digestion (DG) and in vitro duodenal digestion (DD). The antioxidant activity was evaluated using the 1, 1-diphenyl-2-picryl hydrazyl (DPPH), azino-bis-(3-ethylbenzothiazoline-6-sulfonic acid) (ABTS) and oxygen radical absorbance capacity (ORAC) methods. Gastric and duodenal digest of QPI-NG and QPI-G1 and QPI-G3 from white, red and black quinoa presented antioxidant activity. QPI-G1-DD of white quinoa presented the highest antioxidant activity with a DPPH value of 167.98 µmoL TE/g of digest, QPI-G1-DD of red quinoa with an ABTS value of 204.86 µmoL TE/g of digest and QPI-G1-DD of black quinoa with an ORAC value of 401.42 µmoL TE/g of digest. QPI-G3-DD of white quinoa presented higher antioxidant activity with a DPPH value of 186.28 µmoL TE/g of sample, QPI-G3-DD of red quinoa with an ABTS value of 144.06 µmoL TE/g of digest and QPI-G3-DD of black quinoa with an ORAC value of 395.14 µmoL TE/g of digest. The inhibition of reactive oxygen species (ROS) production in the zebrafish embryo model (*Danio rerio*) was evaluated. Protein profiles of QPI from white, red and black from germinated quinoa and non-germinated quinoa were similar with proteins between 10 kDa to 100 kDa with the presence of globulins 11S and 7S and 2S albumins.

## 1. Introduction

Quinoa (*Chenopodium quinoa* Willd) seeds are named Kinua in Quechua indigenous language. Quinoa is a genus of the *Amaranthaceae* family of Andean origin with more than 250 species. Few species are used for human nutrition. The crop is spread around the world in different countries such as the USA, China, Japan, India, UK, Canada, Poland and Australia. Quinoa seeds have a high nutritive value for the presence of phytocomponents. The seeds of quinoa are rich in lipids, carbohydrates, polyphenols, fiber and protein [1,2,3,4]. Protein content is around 12–16% depending on the variety. Proteins have a high nutritive value and quality due to the presence of essential amino acids as leucine and isoleucine. These proteins have a high digestibility as quinoa seeds are free of gluten. Quinoa seeds are used as a diet for celiac people. Quinoa proteins have a high biological value for their biological activities [5,6]. Many biological activities of quinoa proteins have been reported as the antibacterial, antidiabetic, antihypertensive, chemo-preventive, anti-tumoral and antioxidant activities [7,8,9].

The antioxidant activity is evaluated with the degree of inhibition and blocking of free radicals generated by oxidative stress due to the exposure of the organism to harmful substances such as ethanol, peroxides and chemical agents. Chemical phenolic antioxidants such as butylated hydroxyl-anisole (BHA), butylated hydroxyl-toluene (BHT), tertiary butyl-hydroquinone (TBHQ), and propyl gallate (PG) were elaborated in the 1940s to protect petroleum products. At this time, they were used in the food industry to inhibit the oxidation of lipids of food processed as oil and snack products and for other purposes [10,11]. Now, there is interest to find new antioxidant compounds of natural sources such as food proteins and hydrolysates [12,13,14]. The food hydrolysates have an interest in the food industry for different purposes for their biological activities. The European Union in 2018 have approved the use of the hen egg white lysozyme hydrolysate as a novel food ingredient for food supplements for adult people, with the purpose of increasing the blood levels of the amino acid tryptophan. This hydrolysate has a hydrolysis degree between 19–25% DH [15].

Germination is a biological process of the seeds. The process starts with the entry of water in the embryos and finish with the elongation of the embryonic axis. In this process, reserve compounds from embryos such as carbohydrates, lipids and proteins and some other components are degraded to be used in the embryo metabolism. During the embryo development, the levels of anti-nutrient and indigestible components as inhibitor of protease and lectin proteins decrease, the levels of carbohydrates lipids and proteins increase. In the germination process, there are changes in the amino acid composition with the production of intermediate molecular weight peptides [16,17,18,19,20].

In the germination process, secondary phytochemicals can be accumulated as vitamin C and polyphenols compounds [21]. Germinated seeds are new foods in the market with good acceptance for vegetarian people due to the high proportion of carbohydrates, lipids and proteins. Germinated seeds have a high digestibility. Their components have been described with biological activities such as antioxidant, antitumoral, antibacterial and antihypertensive activities [22,23,24]. Germinated seeds can be used to obtain protein concentrates. These protein concentrates from germinated seeds can increase the biological activities after enzymatic hydrolysis. Soybean, legumes, and cereals and pseudo-cereals have been used to obtain germinated seeds with biological activities. For example, a protein isolate from soybean germinated for 72 h presents the capacity to inhibit up to 50% growth of the Hela cell [25]. Robles-Ramírez et al., 2012, described hydrolysates obtained of 6 day germinated soybean presenting anticancer activity with inhibition of 96.5% of growth of cervical cancer cells [26].

Quinoa seeds have been used to produce quinoa protein concentrate. Quinoa protein concentrate (QPC) of (*Chenopodium quinoa* Willd) and Amaranth protein concentrate (APC) of (*Amaranthus caudatus*) have been described to show antioxidant activity and with the capacity to inhibit lipid peroxidation in zebrafish larvae (*Danio rerio*) [27,28]. In these studies, QPC and APC were subject to in vitro gastrointestinal digestion simulation using pepsin and pancreatin enzymes. These hydrolysates presented antioxidant activity.

The aim of this study was to obtain quinoa protein isolates (QPI) of germinated seeds of white, red and black quinoa (*Chenopodium quinoa* Willd), to subject them to in vitro gastrointestinal simulation and finally to evaluate their antioxidant activity and reactive oxygen species (ROS) inhibition in zebrafish embryos (*Danio rerio*).

## 2. Materials and Methods

### 2.1. Materials

Pepsin enzyme [(EC 3.4.23.1), 4500 U/mg], pancreatin from porcine pancreas, mix of enzymes (trypsin, amylase and lipase, ribonuclease, and protease) EC 232-468-9, (10,000 U/mg), salt bile extract, fluorescein disodium (FL), dimethyl sulfoxide (DMSO), and 2,7-dichlorofluorescein diacetate (DCFH-DA) 2, 2′-Azino-bis-(3-ethylbenzothiazoline-6-sulfonic acid) (ABTS), 1, 1-diphenyl-2-picryl hydrazyl (DPPH), and bovine serum albumin (BSA) reactive were used. These materials were obtained from Sigma-Aldrich (St. Louis, MO, USA).

2,2′-azobis (2-methylpropionamide)-dihydrochloride (AAPH), o-phthaldialdehyde (OPA), trichloroacetic acid (TCA), trifluoroacetic acid (TFA) and 6-Hydroxy-2,5,7,8-tetramethylchroman-2-carboxylic acid (Trolox) were obtained from Aldrich (Milwaukee, WI, USA). The rest of the chemicals used were of analytical grade to use for HPLC.

### 2.2. Production of Quinoa Protein Isolates (QPI) from Seeds not Germinated

Quinoa seeds, white, red and black quinoa from Ecuador, *Chenopodium quinoa* Willd. Var. Real came from organic crop. Seeds were washed. Once free of saponins, they were used to produce quinoa flour. QPI was prepared according to the protocol of Acosta et al., 2016, with small modifications.

One hundred grams of quinoa flour was suspended in Milli-Q water (1:10, w/v). The pH was adjusted to 8.0 using a solution of 0.5 M NaOH. The suspension was shacked for 1 h and centrifuged at 8000× *g* for 30 min at 4 °C. The precipitate was removed. The pH of the supernatant was adjusted to pH 4.5 using a 2 N HCl solution. It was centrifuged at 8000× *g* for 30 min at 4 °C. The pH of the precipitate obtained was adjusted to pH 7.0. The samples obtained were lyophilized and kept at −80 °C until further use [29]. The QPI protein content was determined by the bicinchoninic acid assay (BCA) method.

### 2.3. Production of Quinoa Protein Isolates (QPI) from Seeds Germinated

Quinoa seeds (100 g) were submerged in 500 mL of 0.1% sodium hypochlorite solution (1:5, w/v) for 30 min at 25 °C. Then, quinoa seeds were washed with distilled water. The pH was adjusted at pH 7.0. The distilled water was drained and finally, the hydrated seeds were placed in trays on a wet filter paper. Germination was carried out in a sterile plate germination. Germination was performed in darkness at a temperature of 28 °C and at times (24–72 h, named G1 and G3). All germinates were made in triplicate [30]. QPI from germinated seeds was prepared by alkaline extraction at pH 8.0, followed by an isoelectric precipitation at pH 4.5 with the same conditions described in the previous section. The samples obtained were lyophilized and kept at −80 °C until further use. The protein germinated QPI content was determined using the BCA method.

### 2.4. Germinated and Non-Germinated QPI In Vitro Simulated Hydrolysis

QPI-NG and QPI-G1 and QPI-G3 (10 mg/mL) from white, red and black quinoa was subject to a simulated gastric and duodenal digestion using an in vitro model. This method has two phases. Phase 1: Gastric phase; QPI was hydrolyzed using a pepsin enzyme (2000 U/mL) at pH 3.0 for 2 h at 37 °C with agitation. The pepsin enzyme was inactivated by heating at 90 °C for 5 min. Phase 2: Duodenal phase; one milliliter of phase 1 was mixed with one milliliter of pancreatin solution (100 U/mL) at pH 7.0 in the presence of bile salts and Bis-Tris buffer for 2 h at 37 °C. The enzymes activity was stopped by heating at 90 °C for 5 min. The samples were lyophilized and frozen at −80 °C [31].

### 2.5. Determination of the Degree of Hydrolysis by the Orthophthalaldehyde (OPA) Method

The hydrolysis degree (%DH) of gastric and duodenal digest from non-germinated and germinated quinoas was calculated using the OPA method described by Morais et al. (2013).

OPA reagent preparation: 25 mL sodium tetraborate (100 mmoL/L) was mixed with 2.5 mL of 20% (w/v) sodium dodecyl sulfate solution, 40 mg of OPA was dissolved in 1.0 mL of methanol and 100 µL of 2-mercaptoethanol. The volume was adjusted to 50 mL.

Derivatization OPA: 10 µL of the sample was mixed with 3.4 mL of the OPA reagent and the mixture was stored at 25 °C for 2 min. The absorbance was measured at 340 nm. %DH was calculated using the Equation:

%DH = Abs × 1934 × d/c

where Abs is the sample absorbance measure, d is the factor of dilution and c is the concentration of protein in the sample (mg/mL) [32].

### 2.6. ABTS Assay

ABTS was assayed according to Arnao et al. (2001). Two stock solutions were prepared at a concentration of 7.4 mM, ABTS solution A and 2.6 mM potassium per-sulfate (K_2_S_2_O_8_) solution B. The work solution was prepared by mixing 10 mL of solution A and 10 mL of solution B. We allowed them to react for 12 h at 25 °C in dark conditions. The solution was then diluted 1:50 in methanol and the absorbance was measured at 734 nm using a spectrophotometer (Thermo Fisher Scientific Evolution 200 UV/Vis, Waltham, MA USA). The sample (150 μL) was mixed with 2850 μL of ABTS reactive. The mixture was left at 25 °C for 2 h in dark conditions. A blank sample was prepared in the same manner except that methanol was used instead of ABTS. A standard curve of Trolox was used (0–500 µmoL Trolox/L). The curve was stablished (y = 0.477x + 0.6973, R² = 0.9971). Each assay was made in duplicate with three measures (*n = 6*). The antioxidant activity was expressed as μmoL Trolox equivalents (TE)/g sample (QPI or digests) [33].

### 2.7. Oxygen Radical Absorbance Capacity-Fluorescein (ORAC-FL) Assay

The measurement was made using an instrument Multi-Mode Microplate Reader (BioTek, Winooski, VT, USA). Samples and Trolox standards were prepared with 50% acetone solution. All other reagents were prepared in a 75 mmol/L phosphate buffer (pH 7.4). Each well in a 96-well plate contained 20 μmoL/L sample or 50% acetone for blank and 225 μL fluorescein (81.63 nmoL/L). The plate with a cover was incubated for 20 min in 37 °C. An amount of 25 μL AAPH (0.36 moL/L) was added to each well to start reaction, resulting in a final total volume of 280 μL. The fluorescence was recorded every minute for 2 h at 37 °C, where excitation and emission of wavelengths were 485 nm and 528 nm. Standards and samples were performed in triplicate with two experiments. Results were expressed as micromoles of Trolox equivalents (TE) per gram of sample, µmoL TE/g sample (QPI or digests) [34].

### 2.8. DPPH Assay

Non-germinated QPI and germinated QPI from white, red and black quinoa, and their hydrolysates, were used to evaluate the antioxidant activity using the DPPH in vitro method according to Brand-Williams et al. (1995). We measured the decrease of the absorbance at 517 nm spectrophotometrically (spectrum SP-2100UV/SP spectrophotometer, China). The sample was dissolved in 0.1 mL of methanol, then 3.9 mL of a 6 × 10^5^ mol/L methanol of 1,1-diphenyl-2-picryl hydrazyl (DPPH) was added. Trolox was used as the reference standard (0–500 µmoL Trolox/L). The curve was stablished (y = 0.5446x + 0.3949, R² = 0.9804). Each assay was made in duplicate with three measures (*n* = 6) with the value of antioxidant activity being expressed as mg of Trolox equivalents (TE) per 100 g DW [35].

### 2.9. Induction of Stress in Embryos Zebrafish Using AAPH

Zebrafish embryos of 7–9 h post-fertilization (7–9 hpf) were used for the test. These embryos (group = 4 embryos) were transferred to the wells of a 12-well plate using an osmotic embryo medium [E2 1× [15 mM NaCl, 0.5 mM KCl, 1.0 mM CaCl_2_. 2H_2_O, 50 µM Na_2_HPO4, 150 µM KH_2_PO_4_, 10 mM MgSO_4_.7H_2_O, 0.7 mM NaHCO_3_]. An amount of 0.5 mg/L of methylene blue solution dissolved in distilled water containing 1.0 mL of (0.1% DMSO) was added to the medium to drill chorion membrane. An amount of 2.0 mg/mL QPI hydrolysate for 2 h was also added. The groups of embryos were treated with 25 mM (AAPH) or AAPH/QPI hydrolysate for 24 h post-fertilization (24 hpf) [36].

### 2.10. ROS Determination in Zebrafish Embryos

ROS presence in zebrafish embryos without chorion was analyzed using a fluorescent technique (DCFH-DA). The embryos were put in a fish medium with 0.1% DMSO solution to permeabilize the chorion membrane of zebrafish eggs. Zebrafish embryos (3–4 hpf) were treated with gastric and duodenal hydrolysates at a concentration of 2.0 mg/mL. Then, 25 mM AAPH solution was added to the multi well plate and incubated for 2 h at 28 °C. The zebrafish embryos were transferred to 96 well plates, treated with a DCFH-DA solution (2.0 µL/mL). The plates were incubated for 2 h in the dark at 28 °C. After incubation, the zebrafish embryos were rinsed in a fresh embryo medium. The chorion membrane was removed with the help of tweezers. The embryos without chorion were washed with Milli-Q water. The fluorescent image of all zebrafish embryos was taken with the help of a fluorescent microscope (Leica DM1000 LED, Wetzlar, Germany), with coupled Moticam 2000 (Taiwan, China) digital camera [37].

### 2.11. Statistical Analysis

Experimental results in this study are presented as means ± standard deviation (SD), (*n* = 5). Differences between group values were determined using the one-way ANOVA analysis, followed by the Tukey’s test. All tests were considered with statistical differences at *P* < 0.05 and *P* < 0.01 using the software Graph Pad Prism 4.

## 3. Results and Discussion

### 3.1. Percentage of Germination of Seeds Quinoas

**Figure 1:**
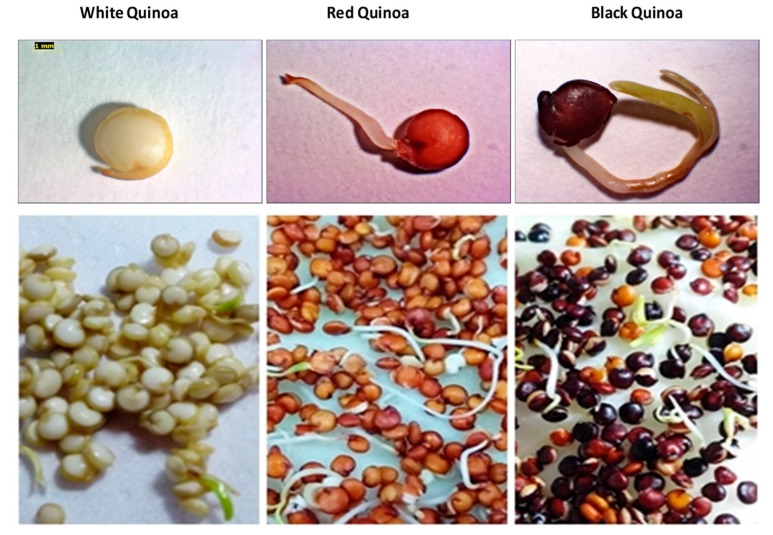
Germinated of white, red and black quinoa (*Chenopodium quinoa* Willd) at 24 h of germination (G1).

**Figure 2:**
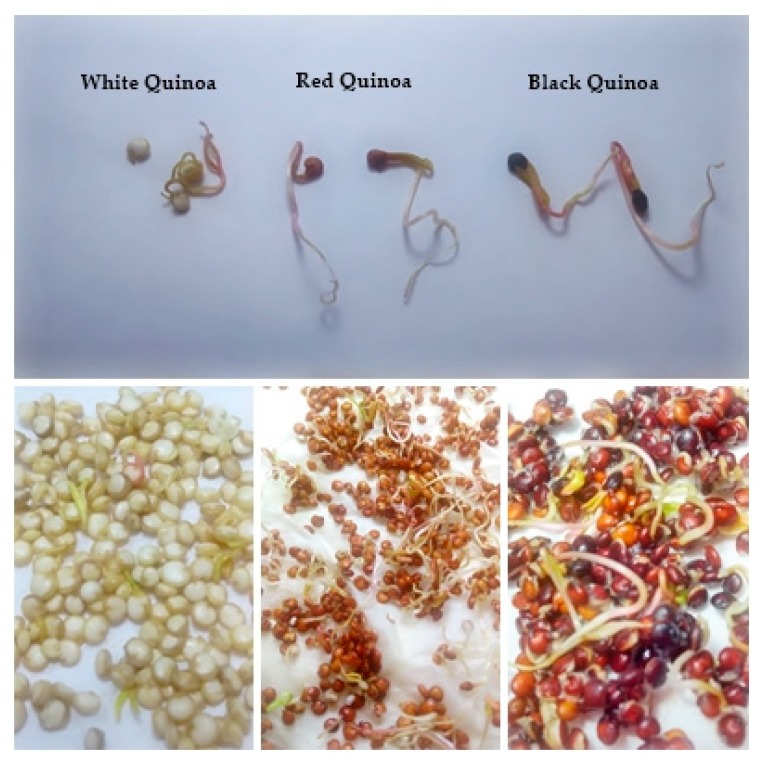
Germinated of white, red and black quinoa (*Chenopodium quinoa* Willd) at 72 h of germination (G3).

Seeds of white, red and black quinoa (*Chenopodium quinoa* Willd) were germinated during 24 h, 48 h and 72 h in the laboratory at 28 °C in darkness. White seeds quinoa presented a retardment in the elongation of radicle assayed at different times (Figure 1). Red and black seeds quinoa presented fast elongation of their radicles and cotyledons. The percentage of germination with the highest value was found in red and black quinoa at 72 h of germination (Figure 2).

**Figure 3:**
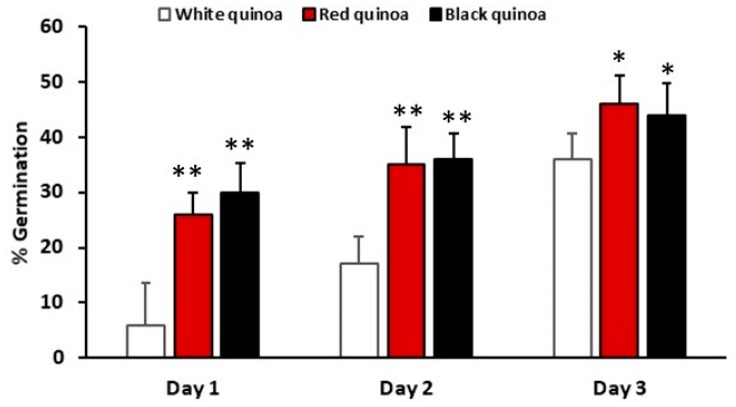
Percentage of germination of white, red and black quinoa (*Chenopodium quinoa* Willd) seeds for 24, 48 and 72 h at 28°C. Means ± (SD), black and red quinoa were compared to white quinoa. Tuckey’s test at ** *P* < 0.05; * *P* < 0.01.

At 24 h of germination, red and black quinoa presented a high percentage of germination, 26% and 30%. At 72 h of germination we found 36% for white quinoa, 46% for red quinoa and 44% for black quinoa (Figure 3). The highest percentage was obtained for red quinoa at 72 h of germination. The germination percentages of white quinoa at 24 h, 48 and 72 h were relativity slow at 28 °C under darkness. Red and black quinoa presented a higher percentage of germination.

The percentage of germination of quinoa seeds was affected by the germination time, and the quinoa variety. Different studies of production of germinated samples of different plants indicate that the percentage of germination depends on time of incubation and temperature. For example, three cultivars of quinoa (*Chenopodium quinoa* Willd) from Iran were used to obtain germinated samples. The results demonstrated that the percentage of germination of quinoa seeds are affected by the cultivar. Each cultivar presented difference in the optimum temperature and time of germination. D’ambrosio et al., 2017, described germination of *Chenopodium quinoa* Willd. Var. Real and *Chenopodium quinoa* Willd. var. Regalona Baer for 4 days of germination with a percentage of germination of 50% and 72% respectively. Our results of germination percentages are in accordance to this study. Red quinoa at 3 days of germination presented 46% of germination. Sajama cultivar at 30 °C for 1 day, presented 80% germination, Santamaría cultivar at 30 °C, for 1 day presented 92% germination and Titicaca cultivar at 30 °C, for 1 day presented 100% germination [38].

### 3.2. Characterization of QPI no Germinated and Germinated Using SDS-PAGE Electrophoresis Analysis

**Figure 4:**
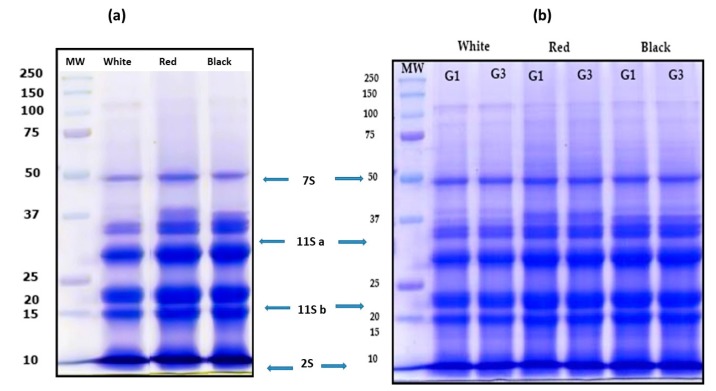
SDS-PAGE analysis of quinoa protein isolate (QPI) obtained from non-germinated and germinated quinoa seeds. (**a**) Profile protein of non-germinated QPI from white, red and black quinoa. (**b**) Profile protein of germinated QPI from white, red and black quinoa. Gel with 12% acrylamide. G1 (1 day of germination) and G3 (3 days of germination).

Germinated QPI and non-germinated QPI of white, red and black quinoa were obtained using an alkaline extraction followed by the isoelectric precipitation method. The pH of isoelectric precipitation was pH 4.5. The protein profile of germinated QPI, non-germinated QPI and its digest were analyzed using the SDS-PAGE electrophoresis analysis. Non-germinated QPI presented a protein profile with bands between 10 kDa at 100 kDa (Figure 4a). In this profile, bands 11S and 7S globulins and 2S albumins can be identified. QPI obtained of seeds germinated (white, red and black quinoa) G1 and G3 present the same profile than non-germinated QPIs with bands between 10 kDa to 100 kDa (Figure 4a). These profiles of proteins were like the profile of QPI proteins from non-germinated seeds.

**Figure 5:**
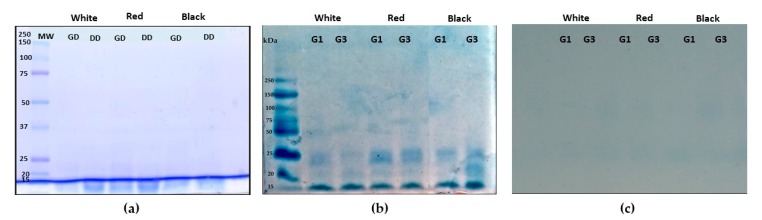
SDS-PAGE analysis of gastric and duodenal digests from non-germinated and germinated white, red and black quinoas. (**a**) Gastric and duodenal digest of QPI from non-germinated quinoa, (**b**) gastric digests of quinoas germinated, (**c**) duodenal digests of white, red and black quinoas germinated, GD (gastric digests), DD (duodenal digest), G1 (1 day of germination) and G3 (3 days of germination).

Figure 5a shows the protein profile of non-germinated QPIs under hydrolysis. QPI-NG from white, red and black quinoa presented the same profile of hydrolysis with pepsin and pepsin/pancreatin. Some proteins bands were not observed in the gel. Only bands were observed in small molecular weights at 15 kDa. These bands correspond to small peptides produced for enzymatic hydrolysis. The gel was made at 12% of concentration of acrylamide.

Figure 5 b shows the profile of gastric hydrolysis of QPIs G1 and G3. This profile was analyzed using the SDS-PAGE technique with 20% of acrylamide. Figure 5b shows a gastric digest profile of non-germinated and germinated QPIs. Germinated and non-germinated QPIs present a similar profile of gastric hydrolysis. Small bands were observed between 25 to 15 kDa. These bands present a better separation, the higher concentration of acrylamide allowed a better separation of the peptides. In duodenal digestion, no bands were observed. The small peptides were not retained by the 20% acrylamide gel. This fact suggests that peptides produced in the duodenal digest are smaller than peptides from the gastric digest (Figure 5c).

QPI protein content from non-germinated and germinated seeds of white, red and black quinoas (*Chenopodium quinoa* Willd) was analyzed using the BCA method. The precipitation isoelectric method allowed us to obtain QPIs. Non-germinated QPIs presented a value of 90.6% of protein content for white quinoa, 94.8% for red quinoa and 95.7% for QPI of black quinoa. QPIs germinated G1 presented values with a high content of protein of 92.4% for QPI-G1 of white quinoa, 107.0% for QPI-G1 of red quinoa and 108.0% for QPI of black quinoa (Table 1). QPIs-G3 presented a lower value than QPIS non-germinated and QPIs-G3. For example, QPI-G3 of black quinoa presented a value of 71.5% of total protein. Shi et al., 2019, described QPI from *Chenopodium quinoa* Willd hydrolyzed with pepsin, papain and pancreatin for 0, 30, 60, 90 and 120 min [39]. They found that %DH increased with the time of hydrolysis. Pepsin enzyme presented a DH high value between 20–35% DH. Our results of digest gastric are in accordance with the %DH values. However, duodenal digest values are higher than the Shi et al., 2019 values [39].

Non-germinated and germinated QPIs were subject to simulated hydrolysis with two phases named gastric digest (GD) with pepsin enzyme and duodenal digest (DD) with pancreatin. Once gastric and duodenal digests were obtained the percentage of hydrolysis (DH) was calculated. The GD samples presented a value of % DH ranging between 17.91% to 32.86% DH (Table 2). The DD samples presented a value of % DH between 22.99 to 64.96% HD. Samples of duodenal digest presented a higher % DH with values of 46.80; 52.35 and 64.96 for white, black and red quinoa, respectively. The sample with the highest value was red quinoa QPI-G3-DD. Pepsin/Pancreatin enzymes produced high hydrolysis of the quinoa proteins. The samples were incubated for 2 h with pepsin and then incubated for 2 h with pancreatin. This combination produced a high % DH in the duodenal digests from quinoas.

Ruiz et al., 2016, described % DH of QPI-E8 hydrolyzed with pepsin for 2 h (*Chenopodium quinoa* Willd) with a value of 14% DH. When QPI-E8 was treated with heat at 60, 90 and 120°C and then hydrolyzed with pepsin, it presented similar values, with %DH of 14, 13 and 12 %DH, respectively. This QPI was fractioned at pH 8.0 and precipitated at pH 5.5 and hydrolyzed in different conditions in the presence of mucin. In both studies, the % DH was determined using the OPA method. We reported % DH for gastric and duodenal digests of non-germinated and germinated QPIs higher than the results reported by Ruiz et al., 2016 [40].

Opazo-Navarrete et al., 2018 reported % DH of QPI (*Chenopodium quinoa* Willd, variety Riobamba) with values of 10.5%. QPI heated at 120 °C and hydrolyzed with pepsin presented a value of 8.5%. Protein-enriched flour and hydrolyzed with pepsin for 180 min presented a value of DH 16%. Our values were higher than the ones of Opazo-Navarrete’s. Our study had different hydrolysis conditions: Time of hydrolysis and pH of hydrolysis. In both studies the % DH was evaluated with the OPA method [41].

Aluko and Monu, 2003, reported a hydrolysate of quinoa obtained using the alcalase enzyme for 4 h of incubation with values of % DH of 48% [42]. Mudgil et al., 2019, described hydrolysates of quinoa (*Chenopodium quinoa* Willd) produced with bromelain, chymotrypsin and protease enzymes for 2, 4 and 6 h of incubation. Chymotrypsin was the enzyme with a high capacity for quinoa proteins hydrolysis. Hydrolysate of 4 and 6 h presented values of 86.0% and 87.05% DH respectively. Bromelain enzyme presented a percentage of hydrolysis of 76.0% at 6 h of incubation. The protease enzyme presented 78% DH at 6 h of incubation [43]. The type of enzyme used, the times of incubation and other hydrolysis parameters can affect the % DH. Hydrolysates from germinated QPI presented a high % DH value. The time of germination process can increase the % DH.

### 3.3. Antioxidant Activity of Non-Germinated and Germinated QPI and their Digest

#### 3.3.1. Evaluation of Antioxidant Activity by the DPPH Method

**Figure 6:**
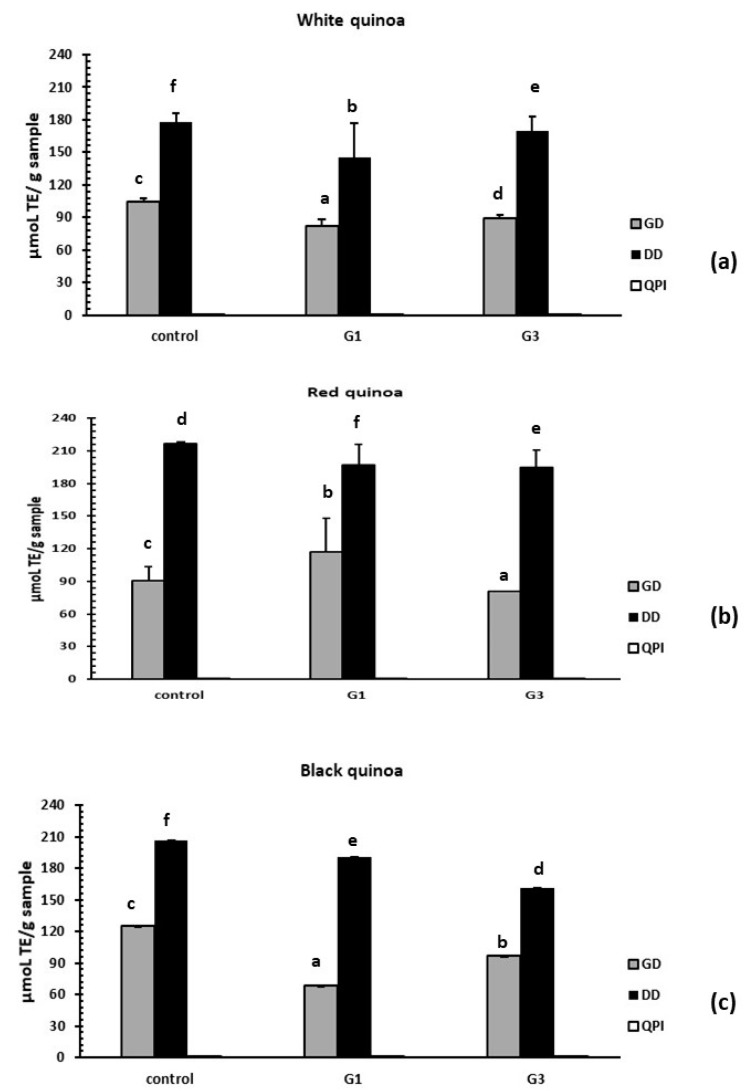
Antioxidant activity of hydrolysates obtained of germinated QPI using 1, 1-diphenyl-2-picryl hydrazyl (DPPH) assay. (**a**) Hydrolysates from germinated white quinoa. (**b**) Hydrolysates from germinated red quinoa. (**c**) Hydrolysates from germinated black quinoa. QPI-NG (QPI non germinated). QPI-G1 (germinated for 1 day) and QPI-G3 (germinated for 3 days). GD (gastric digest). DD (duodenal digest) and QPI (quinoa protein isolate without hydrolysis). Control (QPI of quinoa non-germinated). Results are the means ± standard deviation (SD) of six determinations (*n* = 6). Different superscripts letter for each quinoa cultivar over bars indicate significant statistical differences by one way Anova followed by Tukey’s test (*P* < 0.05).

Non-germinated QPI without hydrolysis from white, red and black quinoa have no antioxidant activity using the DPPH method. Only gastric and duodenal digest from non-germinated QPI and germinated QPI (G1 and G3) were active. QPI-DD non-germinated of white quinoa present antioxidant activity with a value of 187.71 µmoL TE/g of digest, QPI-G1-DD of white quinoa presented a value of 152.26 µmoL TE/g of digest and QPI-G3-DD of white quinoa presented a value of 178.13 µmoL TE/g of digest. Germinated QPI of white quinoa presented a value slightly higher than non-germinated QPI DD of white quinoa. The samples obtained of black seeds quinoas such as QPI-DD of non-germinated black quinoa presented a value of 201.49 µmoL TE/g of digest, B-QPI-G1-DD presented a value of 190.04 µmoL TE/g of digest and QPI-G3-DD of black quinoa presented a value of 150.97 µmoL TE/g of digest. QPI-DD non-germinated red quinoa presented a value of 224.83 µmoL TE/g of digest, QPI-G1-DD of red quinoa presented a value of 198.71 µmoL TE/g of digest and QPI-G3-DD of red quinoa had a value of 190.23 µmoL TE/g of digest. In general, the duodenal digest samples were more active than the gastric digest samples (Figure 6).

#### 3.3.2. Evaluation of Antioxidant Activity by the ABTS Method

**Figure 7:**
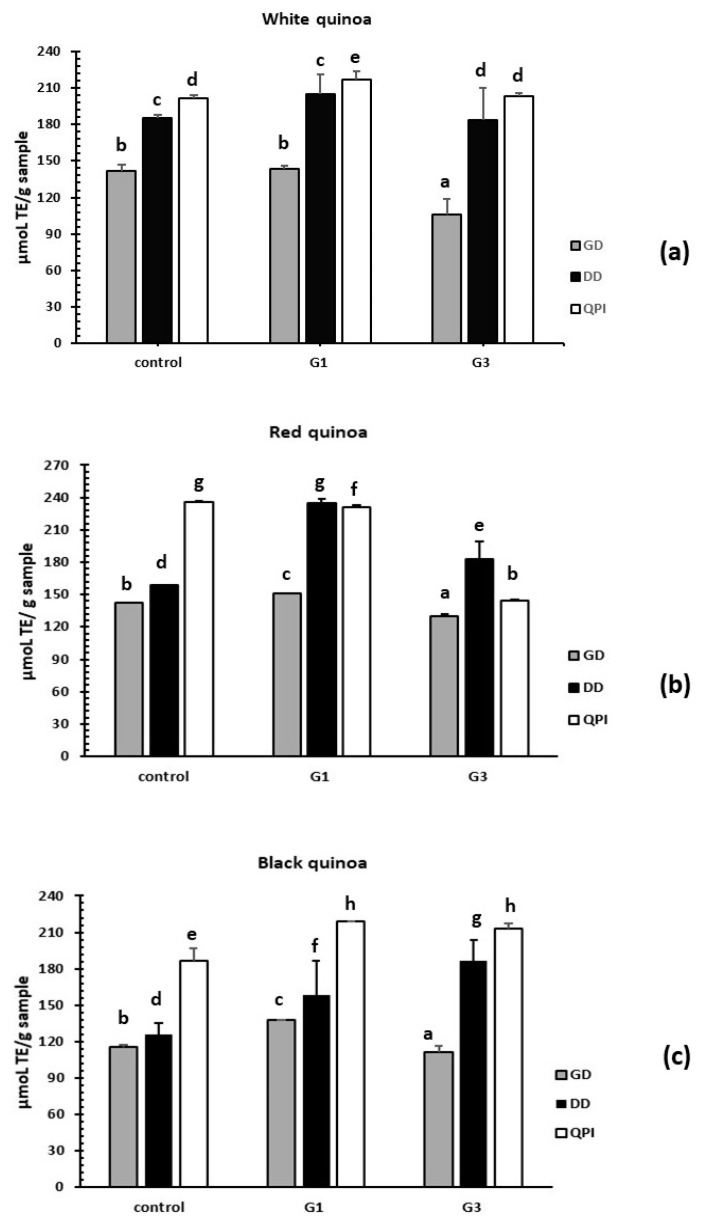
Antioxidant activity of hydrolysates obtained of germinated QPI using azino-bis-(3-ethylbenzothiazoline-6-sulfonic acid) (ABTS) assay. (**a**) Hydrolysates from white quinoa germinated, (**b**) hydrolysates from red quinoa germinated, (**c**) hydrolysates from black quinoa germinated. Control (non-germinated QPI). G1 (germinated for 1 day) and G3 (germinated for 3 days). GD (gastric digestion). DD (duodenal digestion) and QPI (quinoa protein isolate without hydrolysis). Control (QPI of quinoa non-germinated). Results are the means ± standard deviation (SD) of six determinations (*n* = 6). Different superscripts letter for each quinoa cultivar over bars indicate significant statistical differences by one way Anova followed of Tukey’s test (*P* < 0.05).

Non-germinated QPI and germinated QPI and digests from white, red and black quinoa (*Chenopodium quinoa* Willd) were used to evaluate the antioxidant capacity using the ABTS assay. It was observed that the more active samples were non-germinated QPI and germinated QPI without hydrolysis from white, red and black seeds of quinoas. Only QPI G1 duodenal digest of white and red quinoa presented a higher value than non-germinated QPI. QPI-G1 of red quinoa presented a value of 234.71 µmoL TE/g of QPI and red quinoa QPI-NG presented a value of 230.85 µmoL TE/g of QPI. Also, red quinoa QPI-G3 presented a higher value of antioxidant activity with 182.51 µmoL TE/g of QPI than the respective control. When DPPH and ABTS methods results are compared to the duodenal digest of germinated and non-germinated quinoas, results show an increase of the antioxidant activity. In non-germinated QPIs and germinated QPIs without hydrolysis, samples were simply inactive. In both methods, QPI-G1 of red quinoa was the sample with the highest antioxidant activity. The process of germination increases the susceptibility of the proteolytic enzymes for the quinoa proteins. (Figure 7).

#### 3.3.3. Evaluation of Antioxidant Activity by the ORAC-FL Method

The antioxidant activity of non-germinated QPI and germinated QPI from white, red and black quinoa (*Chenopodium quinoa* Willd). Var. Real was evaluated at concentrations of 10, 50 and 200 µmoL. QPI- NG from white, red and black quinoas presented antioxidant activity using the ORAC method. The activity increases with the concentration. The highest activity was obtained at 200 µmoL of sample. For example, QPI-G3 presents 112.33 < 150.93 < 390.13 at concentrations of 10, 50 and 200 µmoL respectively (Table 3). This activity was similar to QPI-G3 of white, red and black quinoa at the same concentrations. For example, QPI-G3 presented 150.93; 149.55 and 152.35 µmoL TE/g of QPI at 50 µmoL, with no statistical differences at *P < 0.05* (Table 3). When concentration increased in the sample, the antioxidant activity increased, the effect was proportional to the concentration (Table 3). The most active sample was the sample assayed at 200 µmoL.

Antioxidant activity of gastric and duodenal of non-germinated QPI and germinated QPI from white, red and black quinoa (*Chenopodium quinoa* Willd Var. Real) was evaluated at concentrations of 10, 50 and 200 µmoL. The antioxidant activity increased with the increase of the concentration. QPI-G1-DD from white, red and black quinoas presented 230.05 < 277.22 < 412.51 µmoL of QPI (Table 4). The statistical analysis indicates that there are differences at *P* < 0.05. When the antioxidant activity of germinated quinoas was compared to the antioxidant activity of non-germinated quinoas, the antioxidant activity increases more than double. It was observed that non-germinated quinoas have lower values than germinated quinoas. For example, QPI-NG of red quinoa at 10 µmoL of sample presented an ORAC value of 112.35 µmoL TE/g of QPI and QPI-G1-DD of red quinoa at 10 µmoL of digest presented an ORAC value of 228.29 µmoL TE/g of digest. Gastric and duodenal digests at the same concentration presented similar antioxidant activity with small differences (Table 4).

Many extracts from *Chenopodium quinoa* Willd were obtained with different solvents such as ethanol, methanol and acetone. These extracts content polyphenols, carotenoids, flavonoids and anthocyanins molecules with high antioxidant activity. This activity has been evaluated using different antioxidant assays such as DPPH, ABTS, FRAP (ferric reducing antioxidant power), PCL (photo chemiluminescence of luminol) and the ORAC method. For example, Tang et al., 2015, have described carotenoids and tocopherols from white, red and black quinoa with antioxidant activity with a value of DPPH of 4.5; 4.8 and 5.8 µmoL TE/g, ORAC of 4.5; 5.7 and 6.5 µmoL TE/g, PCL of 4.5; 4.7 and 5.3 µmoL TE/g and FRAP of 6.40; 7.20 and 7.80 µmoL AAE/g [44].

Jin et al., 2017 reported extract ethanolic obtained from quinoa (*Chenopodium quinoa* Willd) cultivated in Korea, USA and Peru. They reported high antioxidant activity using both the FRAP and DPPH methods. Korean quinoa extract presented a FRAP value of mM/kg of dry material and a DPPH value of 95.29%, USA quinoa extract presented a FRAP value of 8.42 mM/kg of dry material and a DPPH value of 94.50%. Peruvian quinoa extract presented a FRAP value of 7.12 mM/kg of dry material and a DPPH value of 85.16% [45].

Paucar-Menacho et al. (2017) described germinated samples obtained of quinoa (*Chenopodium quinoa* Willd var. INIA-415 Pasankalla) from Perú. They reported that the process of germination induces the accumulation of bioactive compounds such as polyphenols and γ-aminobutyric acid (GABA). Extract methanolic were obtained of germinated quinoa and were used to evaluate the antioxidant activity using the ORAC method. They described that the extract of germinated quinoa increases the antioxidant activity with respect to the extract of non-germinated quinoa. Germinated quinoa presented ORAC values between 315.8–1410.42 mg TE/100 g DW, while quinoa seeds presented a range value of 1085.75 mg TE/100 g DW. The optimum germination conditions were stablished at 20 °C for 42 h. At these conditions they presented a higher TPC content and antioxidant activity [46].

Nowadays, there is an interest to find antioxidant compounds of protein sources. *Chenopodium quinoa* Willd include proteins with interest for their biological properties. Many proteins derived as hydrolysates are evaluated using the in vitro and in vivo antioxidants methods. For example, Vilcacundo et al., 2018, reported gastric and duodenal digest of QPC (quinoa protein concentrate) with antioxidant activity using the ORAC method. QG120 (QPC gastric digest for 120 min) reported an ORAC value of 1.03 µmoL TE/mg of protein and QD120 (QPC duodenal gastric for 120 min) presented an ORAC value of 2.22 µmoL TE/mg of protein. These hydrolysates were fractioned using an ultrafiltration membrane. They found that the fraction with peptides < 5kDa presented high antioxidant activity using the ORAC method [9].

Mudgil et al., 2019, described hydrolysates obtained with bromelain, chymotrypsin and protease with high antioxidant activity using the ABTS and DPPH methods. Hydrolysate of quinoa obtained with chymotrypsin at 2 and 6 h of incubation presented a high ABTS value of 420.5 and 498.6 µmoL TE/µg and a DPPH value of 1442.5 and 1188.0 µmoL TE/µg, respectively [43].

The antioxidant activity results reported in this study are higher than the ones of Mudgil et al., 2019, because our data measures antioxidant activity as µmoL TE/g sample. Pepsin is an efficient enzyme to produce hydrolysates with antioxidant potential at 2 h of incubation at pH 3.0. When pepsin and pancreatin are combined, the antioxidant activity of non-germinated and germinated hydrolyzed QPI increases [43]. The production of small peptides with pepsin and pancreatin increases the % DH in duodenal digests.

Li et al. (2018) described QPI treated with an ultrasound technique (200, 400 and 600 W). QPI was hydrolyzed with a catalase enzyme. The antioxidant activity was evaluated using the DPPH and ABTS methods. QPI-H400 (6 mg/mL) present high antioxidant activity with a value of DPPH of 80% RSA and QPI-H400 (2 mg/mL) present high antioxidant activity using the ABTS method with a value of 80% RSA [47].

Nongonierma et al., 2015 described QPI (quinoa protein isolate), QPH-P (quinoa protein hydrolysate with papain) and QPH-PL (quinoa protein hydrolysate with papain-like) with antioxidant activity using the ORAC method with a value of 264.42; 501.60 and 514.36 µmoL TE/g respectively [48].

The germination process can be a good tool to increase the content of bioactive compounds in food and food-derived from quinoa and increase the biological activities such as the antioxidant activity. Consumers are interested in fresh foods of vegetal sources such as germinated from quinoa, soybean, lentils and lupin. Many vegan supermarkets have as products sprouts of white, red and black quinoas.

### 3.4. Reduction of Stress Oxidative ROS by Germinated QPI Digests

Zebrafish (*Danio rerio*) is an emerging animal experimentation model with many possibilities of use in medicine, pharmacy, molecular biology, and biotechnology. Zebrafish genomic expression for certain diseases is similar to humans [49,50]. DCFH-DA fluorescent assays can be used to evaluate and quantify ROS production in cell lines in an in vivo animal model. Jensen et al. (2016) described PMN cells incubated with DCFH-DA and samples (aqueous cyanophyta extract). The oxidative stress was induced with hydrogen peroxide, the cells with fluorescence intensity were immediately analyzed [51]. Reduction of the intensity of the fluorescence indicated the reduction of ROS in cells. Kang et al. (2014) adapted the DCFH-DA assay to be used in zebrafish embryos. They reported a reduction of ROS in zebrafish embryos when embryos were incubated with polysaccharide molecule isolate of aloe vera (*Aloe barbadensis*) [52].

**Figure 8:**
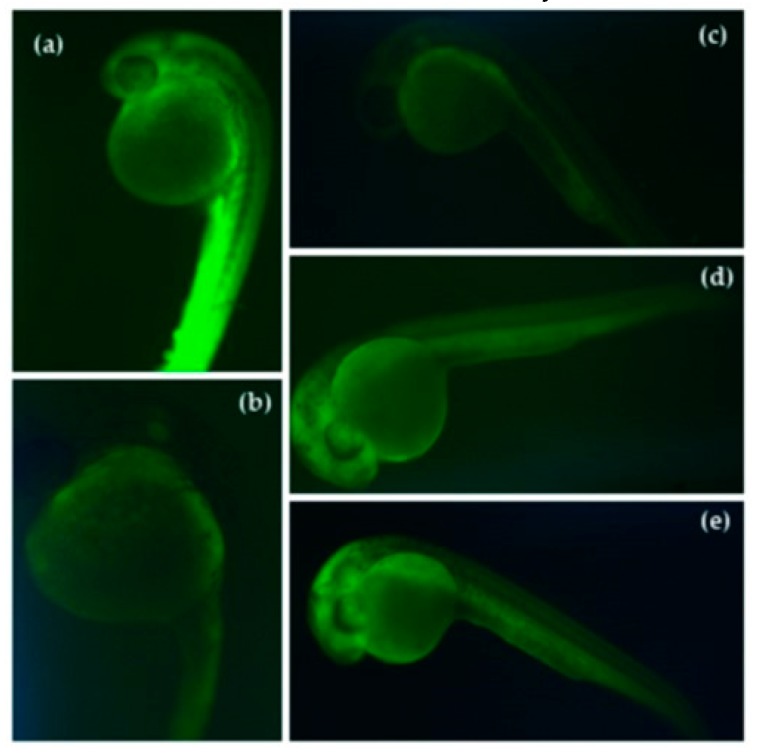
Effects of duodenal digest from germinated QPI for 72 h (G3) against 2,2′-azobis (2-methylpropionamide)-dihydrochloride (AAPH)-induced oxidative stress (ROS) in the zebrafish embryo model. (**a**) AAPH treated group. (**b**) Non-treated group. (**c**) Duodenal digest of white QPI-G3. (**d**) Duodenal digest of red QPI-G3. (**e**) Duodenal digest of black QPI-G3.

Zebrafish embryos without chorion membrane were used to evaluate the reduction of oxidative stress to quantify the reduction of intracellular ROS with the treatment of germinated QPI digests. The groups of embryos were treated with a DCFH-DA fluorescence technique which an intracellularly ROS with the DFC green fluorescent compound (Figure 8a–e). The oxidative stress was induced by the embryos AAPH exposition. A decrease of the green fluorescence in the zebrafish embryos indicated a ROS reduction in zebrafish embryos. This method has been described for different authors using the emergent zebrafish model and cell lines. For example, Figure 8 shows the imagen of zebrafish embryos after the DFCH-DA assay. Figure 8a shows the group of embryos treated with control (AAPH). The intensity of these groups was considered as 100% of ROS production. Embryos treated with AAPH presented strong green fluorescence indicating a high ROS production. Only the group of embryos treated with QPI-G3-DD of white quinoa was able of reducing the intensity of green fluorescence (Figure 8c). Vilcacundo et al., 2018 reported gastric and duodenal digests from *Amaranthus caudatus* with the capacity to reduce the ROS formation in zebrafish embryos [28]. Piñuel et al., 2019 recently described hydrolysates from *Phaseolus vulgaris* with potential to inhibit the ROS formation in zebrafish embryos [31].

## 4. Conclusions

White, red and black quinoa (*Chenopodium quinoa* Willd Var. Real) can be used germinated to produce protein isolate with biological activities, resulting in an increased quality and nutritional value. QPI, obtained from germinated quinoa and treated with pepsin, increases the hydrolysis capacity presenting a high % DH. This gastric and duodenal digest presents a high antioxidant activity. This enzyme produces small fragments of proteins that can be reactive with radicals used in the antioxidants assay. QPI-DD of white quinoa was able of decreasing the ROS formation. We used the in vivo model zebrafish embryos. Germinated quinoa and QPI can be used as new foods for their antioxidant potential and proteins quality.

## Figures and Tables

**Table 1:** Protein content of non-germinated QPI and germinated using the BCA method.

% Protein Content			
Sample	Not Germinated	1 Day of Germination	3 Days of Germination
QPI- white quinoa	90.6 ± 2.55 ^a^	92.4 ± 1.76 ^a^	87.0 ± 0.59 ^a^
QPI- red quinoa	94.8 ± 0.43 ^b^	107.0 ± 1.85 ^b^	91.2 ± 0.75 ^b^
QPI-black quinoa	95.7 ± 0.21 ^b^	108.0 ± 3.68 ^b^	71.5 ± 0.92 ^c^

Results are the means ± standard deviation (SD) of six determinations (*n* = 6). Different superscripts letter for each quinoa cultivar within columns indicate significant statistical differences by one way Anova followed by the Tukey’s test (*P* < 0.05).

**Table 2:** Percentage degree hydrolysis (DH) of gastric and duodenal digest from non-germinated and germinated QPI.

Sample	% DH of Gastric Phase	% DH of Duodenal Phase
**White quinoa**		
QPI-NG	27.44 ± 0.21 ^e^	23.61 ± 107 ^a^
QPI-G1	23.86 ± 1.44 ^d^	22.99 ± 0.80 ^a^
QPI-G3	23.40 ± 0.25 ^d^	46.80 ± 5.10 ^e^
**Black quinoa**		
QPI-NG	19.68 ± 0.29 ^b^	28.14 ± 5.36 ^b^
QPI-G1	28.70 ± 2.31 ^e^	39.96 ± 2.58 ^d^
QPI-G3	27.46 ± 2.14 ^a^	52.35 ± 0.26 ^f^
**Red quinoa**		
QPI-NG	17.91 ± 1.04 ^a^	36.81 ± 0.65 ^c^
QPI-G1	32.86 ± 1.87 ^d^	40.68 ± 2.47 ^d^
QPI-G3	21.19 ± 0.60 ^c^	64.96 ± 3.19 ^g^

QPI (quinoa protein isolate), NG (non-germinated, G1 (1 day of germination) and G3 (3 days of germination). Means ± (SD) (*n* = 3) in a column with different letters (a–g) are significantly different by Tuckey’s test at *P* < 0.05.

**Table 3:** Antioxidant activity of non-germinated and germinated QPIs of white, red and black quinoas using the ORAC method.

ORAC (µmoL TE/g QPI)			
Samples	10 µmoL	50 µmoL	200 µmoL
**White quinoa**			
QPI-NG	112.80 ± 1.12 ^a^	151.56 ± 0.95 ^d^	175.15 ± 1.35 ^b^
QPI-G1	110.01 ± 0.97 ^a^	147.80 ± 0.91 ^a^	170.82 ± 1.83 ^a^
QPI-G3	112.33 ± 0.83 ^a^	150.93 ± 0.89 ^b^	174.43 ± 1.86 ^b^
**Black quinoa**			
QPI-NG	111.18 ± 1.95 ^a^	149.38 ± 1.77 ^b^	172.64 ± 1.78 ^a^
QPI-G1	111.05 ± 1.78 ^a^	149.21 ± 1.75 ^b^	172.44 ± 2.24 ^a^
QPI-G3	111.31 ± 1.14 ^a^	149.55 ± 1.21 ^b^	172.84 ± 2.21 ^a^
**Red quinoa**			
QPI-NG	112.35 ± 1.10 ^a^	152.21 ± 1.20 ^b^	175.91 ± 2.00 ^b^
QPI-G1	113.29 ± 0.96 ^a^	152.21 ± 0.85 ^b^	175.91 ± 2.15 ^b^
QPI-G3	113.47 ± 0.98 ^a^	152.35 ± 0.81 ^b^	175.66 ± 2.23 ^b^

QPI-NG (Non-germinated quinoa protein isolate), QPI-G1 (quinoa protein isolate germinated for 1 day), QPI-G3 (quinoa protein isolate germinated for 3 days. Results are the means ± standard deviation (SD) of six determinations (*n* = 6). Different superscripts letter for each quinoa cultivar within columns indicate significant statistical differences by one way Anova followed of Tukey’s test (*P* < 0.05).

**Table 4:** Antioxidant activity of QPIs digest of non-germinated and germinated white, red and black quinoas using the ORAC method.

ORAC (µmoL TE/g Digests)			
Samples	10 µmoL	100 µmoL	200 µmoL
**White quinoa**			
QPI-NG-GD	181.62 ± 3.22	233.85 ± 3.00	280.10 ± 1.78
QPI-NG-DD	206.23 ± 4.15	260.36 ± 3.42	345.91 ± 1.02
QPI-G1-GD	190.01 ± 2.36	240.70 ± 2.08	305.22 ± 2.07
QPI-G1-DD	220.01 ± 0.57	275.01 ± 4.15	394.62 ± 3.19
QPI-G3-GD	195.08 ± 3.45	252.87 ± 2.09	310.13 ± 4.11
QPI-G3-DD	224.56 ± 2.13	272.87 ± 6.26	390.13 ± 5.03
**Black quinoa**			
QPI-NG-GD	184.89 ± 1.09	237.94 ± 3.36	275.43 ± 5.47
QPI-NG-DD	200.14 ± 4.03	257.11 ± 1.09	352.67 ± 3.12
QPI-G1-GD	230.05 ± 3.18	278.22 ± 1.55	401.42 ± 2.36
QPI-G1-DD	230.05 ± 3.18	277.22 ± 3.44	412.51 ± 3.98
QPI-G3-GD	235.31 ± 2.00	279.24 ± 0.60	395.14 ± 4.023
QPI-G3-DD	228.35 ± 2.30	273.01 ± 0.31	385.91 ± 2.25
**Red quinoa**			
QPI-NG-GD	180.02 ± 1.18	241.95 ± 0.01	272.11 ± 2.55
QPI-NG-DD	203.55 ± 1.10	263.08 ± 0.35	349.11 ± 1.00
QPI-G1-GD	232.05 ± 1.95	281.54 ± 0.94	396.21 ± 2.37
QPI-G1-DD	229.28 ± 3.24	295.63 ± 1.92	398.55 ± 2.60
QPI-G3-GD	233.35 ± 3.19	290.64 ± 1.11	385.04 ± 3.00
QPI-G3-DD	228.29 ± 2.65	275.49 ± 3.34	382.09 ± 3.14

QPI-NG (non-germinated quinoa protein isolate), QPI-G1 (quinoa protein isolate obtained of quinoa germinated for 1 day), QPI-G3 (quinoa protein isolate obtained of quinoa germinated for 3 days). GD (gastric digests) and DD (duodenal digests). Results are the means ± standard deviation (SD) of six determinations (*n* = 6). Different superscripts letter for each quinoa cultivar within columns indicate significant statistical differences by one way Anova followed of Tukey’s test (*P* < 0.05).

## References

[1] Vilcacundo R., Hernández-Ledesma B. (2017). Nutritional and biological value of quinoa (*Chenopodium quinoa* Willd). Curr. Opin. Food Sci..

[2] Pellegrini M., Lucas-Gonzales R., Ricci A., Fontecha J., Fernández-López J., Pérez-Álvarez J.A., Viuda-Martos M. (2018). Chemical, fatty acid, polyphenolic profile, techno-functional and antioxidant properties of flours obtained from quinoa (*Chenopodium quinoa* Willd) seeds. Ind. Crops Prod..

[3] Vega-Gálvez A., Miranda M., Vergara J., Uribe E., Puente L., Martínez E.A. (2010). Nutrition facts and functional potential of quinoa (*Chenopodium quinoa* Willd), an ancient Andean grain: A review. J. Sci. Food Agric..

[4] Jaikishun S., Li W., Yang Z., Song S. (2019). Quinoa: In perspective of global challenges. Agronomy.

[5] Toapanta A., Carpio C., Vilcacundo R., Carrillo W. (2016). Analysis of protein isolate from quinoa (*Chenopodium quinoa* Willd). Asian J. Pharm. Clin. Res..

[6] Abugoch L.E., Romero N., Tapia C.A., Silva J., Rivera M. (2008). Study of some physicochemical and functional properties of quinoa (*Chenopodium quinoa* Willd) protein isolates. J. Agric. Food Chem..

[7] Hu Y., Zhang J., Zou L., Fu C., Li P., Zhao G. (2017). Chemical characterization, antioxidant, immune-regulating and anticancer activities of a novel bioactive polysaccharide from *Chenopodium quinoa* seeds. Int. J. Biol. Macromol..

[8] Vilcacundo R., Martínez-Villaluenga C., Hernández-Ledesma B. (2017). Release of dipeptidyl peptidase IV. α-amylase and α-glucosidase inhibitory peptides from quinoa (*Chenopodium quinoa* Willd.) during *in vitro* simulated gastrointestinal digestion. J. Funct. Foods.

[9] Vilcacundo R., Miralles B., Carrillo W. (2018). Hernández-Ledesma B in vitro chemopreventive properties of peptides released from quinoa (*Chenopodium quinoa* Willd) protein under simulated gastrointestinal digestion. Food Res. Int..

[10] Saito M., Sakagami H., Fujisawa S. (2003). Cytotoxicity and apoptosis induction by butylated hydroxyanisole (BHA) and butylated hydroxytoluene (BHT). Anticancer Res..

[11] Nieva-Echevarría B., Manzanos M.J., Goicoechea E., Guillén M.D. (2015). 2, 6-Di-tert-butyl-hydroxytoluene and its metabolites in foods. Compr. Rev. Food Sci. Food Saf..

[12] Carrillo W., Ramos M. (2018). Identification of antimicrobial peptides of native and heated hydrolysates from hen egg white lysozyme. J. Med. Food.

[13] Carrillo W., Gómez-Ruiz J.A., Miralles B., Ramos M., Barrio D., Recio I. (2016). Identification of antioxidant peptides of hen egg-white lysozyme and evaluation of inhibition of lipid peroxidation and cytotoxicity in the Zebrafish model. Eur. Food Res. Technol..

[14] Rodríguez Saint-Jean S., De las Heras A., Carrillo W., Recio I., Ortiz-Delgado J.B., Ramos M., Pérez-Prieto S.I. (2013). Antiviral activity of casein and αs_2_ casein hydrolysates against the infectious hematopoietic necrosis virus. a rhabdovirus from salmonid fish. J. Fish Dis..

[15] Vilcacundo R., Méndez P., Reyes W., Romero H., Pinto A., Carrillo W. (2018). Antibacterial activity of hen egg white lysozyme denatured by thermal and chemical treatments. Sci. Pharm..

[16] Yang Q.Q., Cheng L., Long Z.-Y., Li H.B., Gunaratne A., Gan R.-Y., Corke H. (2019). Comparison of the phenolic profiles of soaked and germinated peanut cultivars via UPLC-QTOF-MS. Antioxidants.

[17] López-Amorós M.L., Hernández T., Estrella I. (2006). Effect of germination on legume phenolic compounds and their antioxidant activity. J. Food Comp. Anal..

[18] Shi H., Nam P.K., Ma Y. (2010). Comprehensive profiling of isoflavones, phytosterols, tocopherols, minerals, crude protein, lipid, and sugar during soybean (*Glycine max*) germination. J. Agric. Food Chem..

[19] Wang K.H., Lai Y.H., Chang J.C., Ko T.F., Shyu S.L., Chiou R.Y.Y. (2005). Germination of peanut kernels to enhance resveratrol biosynthesis and prepare sprouts as a functional vegetable. J. Agric. Food Chem..

[20] Aguilera Y., Díaz M.F., Jiménez T., Benítez V., Herrera T., Cuadrado C., Martín-Cabrejas M.A. (2013). Changes in non-nutritional factors and antioxidant activity during germination of nonconventional legumes. J. Agric. Food Chem..

[21] Gan R.Y., Wang M.F., Lui W.Y., Wu K., Corke H. (2016). Dynamic changes in phytochemical composition and antioxidant capacity in green and black mung bean (*Vigna radiata*) sprouts. Int. J. Food Sci. Technol..

[22] Carciochi R.A., Manrique G.D., Dimitrov K. (2014). Changes in phenolic composition and antioxidant activity during germination of quinoa seeds (*Chenopodium quinoa* Willd). Int. Food Res. J..

[23] Mamilla R.K. (2017). Effect of germination on antioxidant and ACE inhibitory activities of legumes. LWT-Food Sci. Technol..

[24] De Souza Rocha T., Hernandez L.M.R., Mojica L., Johnson M.H., Chang Y.K., de Mejía E.G. (2015). Germination of Phaseolus vulgaris and alcalase hydrolysis of its proteins produced bioactive peptides capable of improving markers related to type-2 diabetes in vitro. Food Res. Int..

[25] Wilson K.A., Rightmire B.R., Chen J.C., Tan-Wilson A.L. (1986). Differential proteolysis of glycinin and β-conglycinin polypeptides during soybean germination and seedling growth. Plant Physiol..

[26] Robles-Ramírez M.C., Ramón-Gallegos E., Mora-Escobedo R., Torres-Torres N. (2012). A peptide fraction from germinated soybean protein down-regulates PTTG1 and TOP2A mRNA expression, inducing apoptosis in cervical cancer cells. J. Exp. Ther. Oncol..

[27] Vilcacundo R., Barrio D., Carpio C., García-Ruiz A., Rúales J., Hernández-Ledesma B., Carrillo W. (2017). Digestibility of quinoa (*Chenopodium quinoa* Willd) protein concentrate and its potential to inhibit lipid peroxidation in the Zebrafish larvae model. Plant Foods Hum. Nutr..

[28] Vilcacundo R., Barrio D.A., Piñuel L., Boeri P., Tombari A., Welbaum J., Pinto A., Hernández-Ledesma B., Carrillo W. (2018). Inhibition of lipid peroxidation of kiwicha (*Amaranthus caudatus*) hydrolyzed protein using zebrafish larvae and embryos. Plants.

[29] Acosta C., Carpio C., Vilcacundo R., Carrillo W. (2016). Identification of proteins isolate from amaranth (*Amaranthus caudatus*) by sodium dodecyl sulfate-polyacrylamide gel electrophoresis with water and NaCl 0.1 m solvents. Asian J. Pharm. Clin. Res..

[30] González-Montoya M., Hernández-Ledesma B., Silván J.M., Mora-Escobedo R., Martínez-Villaluenga C. (2018). Peptides derived from in vitro gastrointestinal digestion of germinated soybean proteins inhibit human colon cancer cells proliferation and inflammation. Food Chem..

[31] Piñuel L., Vilcacundo E., Boeri P., Barrio D.A., Morales D., Pinto A., Morán R., Samaniego I., Carrillo W. (2019). Extraction of protein concentrate from red bean (*Phaseolus vulgaris* L.): Antioxidant activity and inhibition of lipid peroxidation. J. Appl. Pharm. Sci..

[32] Morais H.A., Silvestre M.P.C., Silveira J.N., Silva V.D.M., Silva M.R. (2013). Action of a pancreatin and an *Aspergillus oryzae* protease on whey proteins: Correlation among the methods of analysis of the enzymatic hydrolysates. Braz. Arch. Biol. Technol..

[33] Arnao M.B., Cano A., Acosta M. (2001). The hydrophilic and lipophilic contribution to total antioxidant activity. Food Chem..

[34] Moore J., Hao Z., Zhou K., Luther M., Costa J., Yu L. (2005). Carotenoid, tocopherol, phenolic acid, and antioxidant properties of Maryland-grown soft wheat. J. Agric. Food Chem..

[35] Brand-Williams W., Cuvelier M.E., Berset C.L.W.T. (1995). Use of a free radical method to evaluate antioxidant activity. LWT-Food Sci. Technol..

[36] Cunliffe V.T. (2003). Zebrafish: A practical approach. Edited by Nüsslein-Volhard C and Dahm R Oxford university press. Genet. Res. Camb..

[37] Rosenkranz A.R., Schmaldienst S., Stuhlmeier K.M., Chen W., Knapp W., Zlabinger G.P. (1992). A microplate assay for the detection of oxidative product using 2%, 7%-dichlorofluorescein-diacetate. J. Immunol. Met..

[38] D’ambrosio T., Amodio M.L., Pastore D., De Santis G., Colelli G. (2017). Chemical, physical and sensorial characterization of fresh Quinoa Sprouts (*Chenopodium quinoa* Willd) and effects of modified atmosphere packaging on quality during cold storage. Food Packag. Shelf..

[39] Shi Z., Hao Y., Teng C., Yao Y., Ren G. (2019). Functional properties and adipogenesis inhibitory activity of protein hydrolysates from quinoa (*Chenopodium quinoa* Willd). Food Sci. Nutr..

[40] Ruiz G.A., Opazo-Navarrete M., Meurs M., Minor M., Sala G., van Boekel M., Janssen A.E. (2016). Denaturation and in vitro gastric digestion of heat-treated quinoa protein isolates obtained at various extraction pH. Food Biophys..

[41] Opazo-Navarrete M., Freire D.T., Boom R.M., Janssen A E. (2019). The Influence of starch and fibre on in vitro protein digestibility of dry fractionated quinoa seed (Riobamba Variety). Food Biophys..

[42] Aluko R.E., Monu E. (2003). Functional and bioactive properties of quinoa seed protein hydrolysates. J. Food Sci..

[43] Mudgil P., Omar L.S., Kamal H., Kilari B.P., Maqsood S. (2019). Multi-functional bioactive properties of intact and enzymatically hydrolysed quinoa and amaranth proteins. LWT Food Sci. Technol..

[44] Tang Y., Li X., Chen P.X., Zhang B., Hernandez M., Zhang H., Tsao R. (2015). Characterisation of fatty acid, carotenoid, tocopherol/tocotrienol compositions and antioxidant activities in seeds of three Chenopodium quinoa Willd genotypes. Food Chem..

[45] Jin H., Likang Q., Qingnan S., Anyan W. (2017). Effect of quinoa saponins extraction and sprouting on saponins content. J. Chin. Cereals Oils Assoc..

[46] Paucar-Menacho L.M., Martínez-Villaluenga C., Dueñas M., Frias J., Peñas E. (2018). Response surface optimisation of germination conditions to improve the accumulation of bioactive compounds and the antioxidant activity in quinoa. Int. J. Food Sci. Technol..

[47] Li X., Da S., Li C., Xue F., Zang T. (2018). Effects of high-intensity ultrasound pretreatment with different levels of power output on the antioxidant properties of alcalase hydrolyzates from Quinoa (*Chenopodium quinoa* Willd) protein isolate. Cereal Chem..

[48] Nongonierma A.B., Le Maux S., Dubrulle C., Barre C., FitzGerald R.J. (2015). Quinoa (*Chenopodium quinoa* Willd.) protein hydrolysates with in vitro dipeptidyl peptidase IV (DPP-IV) inhibitory and antioxidant properties. J. Cereal Sci..

[49] Engeszer R.E., Patterson L.B., Rao A.A., Parichy D.M. (2007). Zebrafish in the wild: A review of natural history and new notes from the field. Zebrafish.

[50] De Esch C., Slieker R., Wolterbeek A., Woutersen R., de Groot D. (2012). Zebrafish as potential model for developmental neurotoxicity testing: A mini review. Neurotoxicol. Teratol..

[51] Jensen G.S., Attridge V.L., Beaman J.L., Guthrie J., Ehmann A., Benson K.F. (2015). Antioxidant and anti-inflammatory properties of an aqueous *Cyanophyta* extract derived from *Arthrospira platensis*: Contribution to bioactivities by the non-phycocyanin aqueous fraction. J. Med. Food..

[52] Kang M.C., Kim S.Y., Kim Y.T., Kim E.A., Lee S.H., Ko S.C., Jang H.S. (2014). In Vitro and in vivo antioxidant activities of polysaccharide purified from aloe vera (*Aloe barbadensis*) gel. Carbohydr. Polym..

